# HDAC inhibitors enhance the anti-tumor effect of immunotherapies in hepatocellular carcinoma

**DOI:** 10.3389/fimmu.2023.1170207

**Published:** 2023-05-26

**Authors:** Chen Shen, Mei Li, Yujuan Duan, Xin Jiang, Xiaoming Hou, Fulai Xue, Yinan Zhang, Yao Luo

**Affiliations:** ^1^ Department of Laboratory Medicine, Medical Equipment Innovation Research Center/Medical Device Regulatory Research and Evaluation Center, West China Hospital, Sichuan University, Chengdu, China; ^2^ School of Chemical Science and Engineering, Tongji University, Shanghai, China; ^3^ School of Chemistry and Chemical Engineering, Shanghai Jiao Tong University, Shanghai, China

**Keywords:** hepatocellular carcinoma, tumor immune microenvironment, immunotherapy, immune checkpoint inhibitors, HDAC inhibitors, nano-based drug delivery system

## Abstract

Hepatocellular carcinoma (HCC), the most common liver malignancy with a poor prognosis and increasing incidence, remains a serious health problem worldwide. Immunotherapy has been described as one of the ideal ways to treat HCC and is transforming patient management. However, the occurrence of immunotherapy resistance still prevents some patients from benefiting from current immunotherapies. Recent studies have shown that histone deacetylase inhibitors (HDACis) can enhance the efficacy of immunotherapy in a variety of tumors, including HCC. In this review, we present current knowledge and recent advances in immunotherapy-based and HDACi-based therapies for HCC. We highlight the fundamental dynamics of synergies between immunotherapies and HDACis, further detailing current efforts to translate this knowledge into clinical benefits. In addition, we explored the possibility of nano-based drug delivery system (NDDS) as a novel strategy to enhance HCC treatment.

## 1 Introduction

Primary liver cancer is currently the sixth most commonly diagnosed cancer and the third leading cause of cancer-related death worldwide, hepatocellular carcinoma (HCC) accounts for approximately 75%-85% of liver cancer cases (1, 2). Due to the tumor heterogeneity, tumor metastasis, and resistance to traditional chemotherapeutic agents, current treatment options such as surgical resection, radiofrequency ablation, neoadjuvant chemoradiotherapy, and liver transplantation for HCC will only benefit a few percentages of patients, novel therapeutic modalities are urgently needed for patients with advanced or unresectable HCC (3).

The crucial role of the immune system in suppressing the growth, proliferation, and progression of tumors is widely accepted (4). The immunotherapy of tumors mainly utilizes the host immune system to fight the tumor by regulating the host’s own immune function or enhancing the immunogenicity of the tumors (5). HCC is considered to be inflammation-induced cancer, showing good sensitivity to immunotherapies (6). Immunotherapy strategies for HCC mainly include immune checkpoint inhibitors (ICIs), cell-based therapies, and tumor immune vaccines. Cytokines such as interferon also show certain anti-HCC effects (7). Checkpoint inhibitors are typically monoclonal antibodies that target programmed cell death protein 1 (PD-1), programmed death-ligand 1 (PD-L1) or cytotoxic T lymphocyte-associated antigen 4 (CTLA-4). PD-1 is a surface receptor highly expressed by activated T cells, B cells, dendritic cells (DC), and natural killer cells (NK) which provides inhibitory signals to the immune system to modulate the activity of immune cells in peripheral tissues and keep T-cells from attacking normal cells in the body. The interaction between PD-L1 expressed on cancer cells and PD-1 is a key mediator of cancer immune escape, which leads to the suppression of anticancer immunity and the promotion of tumor progression (8). Immune checkpoints blockade with anti-PD-1/PD-L1 antibodies have been successfully utilized in the treatment of various cancers such as melanoma (9), non–small cell lung cancer (10), bladder carcinoma (11), Hodgkin’s lymphoma (12), and Merkel cell carcinoma (13). CTLA-4, another important ICIs target, competitively inhibits the binding of the B7 ligand to the costimulatory receptor CD28, resulting in decreased peripheral T-cell activity. Specific blocking of CTLA-4 can increase the T-cell infiltration of tumors and enhance the killing effect of the immune system on tumors (14, 15). In addition, chimeric antigen receptor T cells (CAR-T) and other cell therapies as well as HCC tumor immune vaccines also show good effects and application prospects. However, the unique inhibitory tumor microenvironment (TME) of HCC and the genetic differences of the host make existing immunotherapies challenges. Compared to unprecedented and durable responses in these T cell-inflamed cancers, the objective response rates (ORRs) of PD-1 and PD-L1 blockade in HCC remain relatively low (16–18). It was proved that TME, specific receptors, and signaling pathways can affect the clinical outcome of PD-1/PD-L1 treatment (19), Combining different immunotherapies or combining immunotherapies with other modalities may provide synergistic effects and facilitate the development of the treatment of HCC (20).

Regulated by related histone-modifying enzymes (HMEs), various post-translational modifications (PTMs) of histone substrates, such as acetylation, methylation, phosphorylation, ubiquitination, and ADP ribosylation, play a crucial role in chromatin dynamics, relative gene regulation and many other biological functions (21). Increasing evidence indicates that abnormal epigenetic regulation of gene transcription associated with histone modifications plays a crucial role in cancer initiation, progression, and metastasis (22). In contrast to direct mutations or deletions in the main DNA sequence, aberrant epigenetic modifications are potentially reversible by epigenetic therapies (23). Several small-molecule inhibitors of HME, such as histone methylation inhibitors, histone demethylation inhibitors, histone deacetylation inhibitors, and DNA methylation inhibitors, can lead to the programmed death of tumor cells by affecting the cell cycle, angiogenesis, proliferation, and migration (24–26). To date, histone deacetylation inhibitors (HDACis) including vorinostat, romidepsin, belinostat, and panobinostat have been approved by FDA for the treatment of hematological malignancies such as cutaneous T-cell lymphoma (CTCL) and multiple myeloma (27–29). Despite promising results in the treatment of blood cancers, the therapeutic efficacy of several HDACis as a single therapeutic agent in solid tumors such as HCC has been unsatisfactory, and the prevalence of drug-induced side effects was relatively high (30). Till now, numerous combination therapies involving HDACis in synergy with chemotherapy, radiotherapy, phototherapy, targeted therapy, and immunotherapy have been efficiently developed to enhance therapeutic efficacy (31).

HDACis can regulate gene expression by regulating host epigenetic modification, thereby overcoming the tolerance of HCC patients to immunotherapy and enhancing the therapeutic effect. HDACis have been shown to promote immunotherapies in a variety of tumors (32). This effect is mainly achieved by enhancing the immunogenicity of the tumor and regulating the tumor immune microenvironment. Studies have shown that HDACis can increase the expression of PD-1/PD-L1, thereby increasing the sensitivity of tumors to ICIs treatment (33). In some tumors, HDACis also increase the expression of MHC molecules that assist the host immune system in recognizing tumor antigens (34). The regulation of HDACis on TME can promote the recruitment of T cells and NK cells and exert the function of tumor inhibition by increasing the expression of chemokines, cytokines and NK cell-related receptors. Similar mechanisms were also found in HCC. Moreover, these mechanisms work together to promote the effect of immunotherapies. The effect of HDACis on immunotherapy also allows these drugs to work without high doses. This reduces the possible cytotoxicity and adverse reactions of immune drugs, and also creates chances for wider research and application (35).

In the past few years, the rapid development of nanotechnology and its application in many fields have had a profound impact on the development of biomedicine (36). Nano-based drug delivery system (NDDS) constructed on the basis of nanomaterials provides an effective and powerful new strategy for enhancing the efficacy of immunotherapy drugs for HCC (37). NDDS specifically targets tumor cells through advanced delivery systems, overcoming inhibitory TME while effectively reducing the damage to normal cells. Currently, a large number of nanomedicine-based therapies are being developed for HCC (38).

Combined multidrug approaches for cancer treatment could overcome the limitations of single therapies, increase antitumor effects, and reduce drug resistance. In this review, we describe immunotherapies and HDACis in detail, explain the mechanism of their therapeutic effects in HCC respectively, and discuss current progress in the combination of novel immunotherapies with HDACis. In addition, concerned that the nano-based drug delivery system (NDDS) exhibits outstanding properties such as targeted delivery, TME response, and site-specific release in the delivery of multi-drug combination, we further discuss the potential clinical applications of NDDS in dual-therapy for HCC briefly.

## 2 Immunotherapy for HCC

### 2.1 The immune microenvironment of HCC

The TME is the environment around a tumor mass that consists not only of a heterogeneous population of cancer cells but also of stromal cells, neovessels, immune cells, and extracellular matrix (ECM). Considering the close relationship and constant interaction between tumors and their surrounding microenvironment, it is becoming increasingly apparent that TME has a significant impact on tumorigenesis, immune evasion, recurrence, as well as drug resistance (39).

The immunosuppressive microenvironment in HCC is thought to be counterbalanced by cells that generate antitumor immune responses and/or clear tumor cells. Liver cells are normally exposed to a significant number of bacterial antigens from portal circulation, leading to constant immune stimulation and antigen exposure. As a result, the liver has developed intrinsic tolerogenic mechanisms in the innate and adaptive immune responses to prevent autoimmune responses and unnecessary tissue damage, which makes it considered an immune-tolerant tissue (40). The immune microenvironment in the liver is dominated by immunosuppressive cells and signals. The key immune suppressor cells implicated in HCC immune escape comprise tissue-resident macrophages (mostly Kupffer cells), regulatory T (T_reg_) cells, and myeloid suppressor cells (MDSCs) (41, 42). Known as specialized macrophages located in the liver, Kupffer cells remove bacteria and produce immunosuppressive cytokines, such as IL-10 and prostaglandins. Additionally, they are capable of negatively regulating immune response by expressing the inhibitory immune checkpoint ligand PD-L1, recruiting T_reg_ cells, and IL-17-expressing CD4^+^ T helper 17 (TH17) cells, as well as downregulating major histocompatibility complex class II (MHC II) and costimulatory molecules (41–43). T_reg_ cells and monocyte-derived tumor-associated macrophages (TAMs) can suppress innate and adaptive immunity against HCC through the cooperation with dysfunctional DCs, dysfunctional CD8^+^PD-1^+^ T cells, neutrophils, and regulatory B (B_reg_) cells (43–45). The high numbers of MDSCs in the liver produce vascular endothelial growth factor (VEGF), transforming growth factor-β (TGF-β), and arginase, which also suppress T cell activation (41). There is a higher abundance of T_reg_ cells and MDSCs in peripheral blood among HCC patients than in normal individuals (46, 47).

The deepening of research and the development of technology have improved our understanding of the complexity and heterogeneity of the tumor immune microenvironment and its components, and their effects on response to tumor immunotherapy. Tumor immunotherapy is considered to be a novel and promising therapy for tumors and it has recently become a hot research topic.

### 2.2 Immunotherapies and immune checkpoint inhibitors for HCC

**Figure 1:**
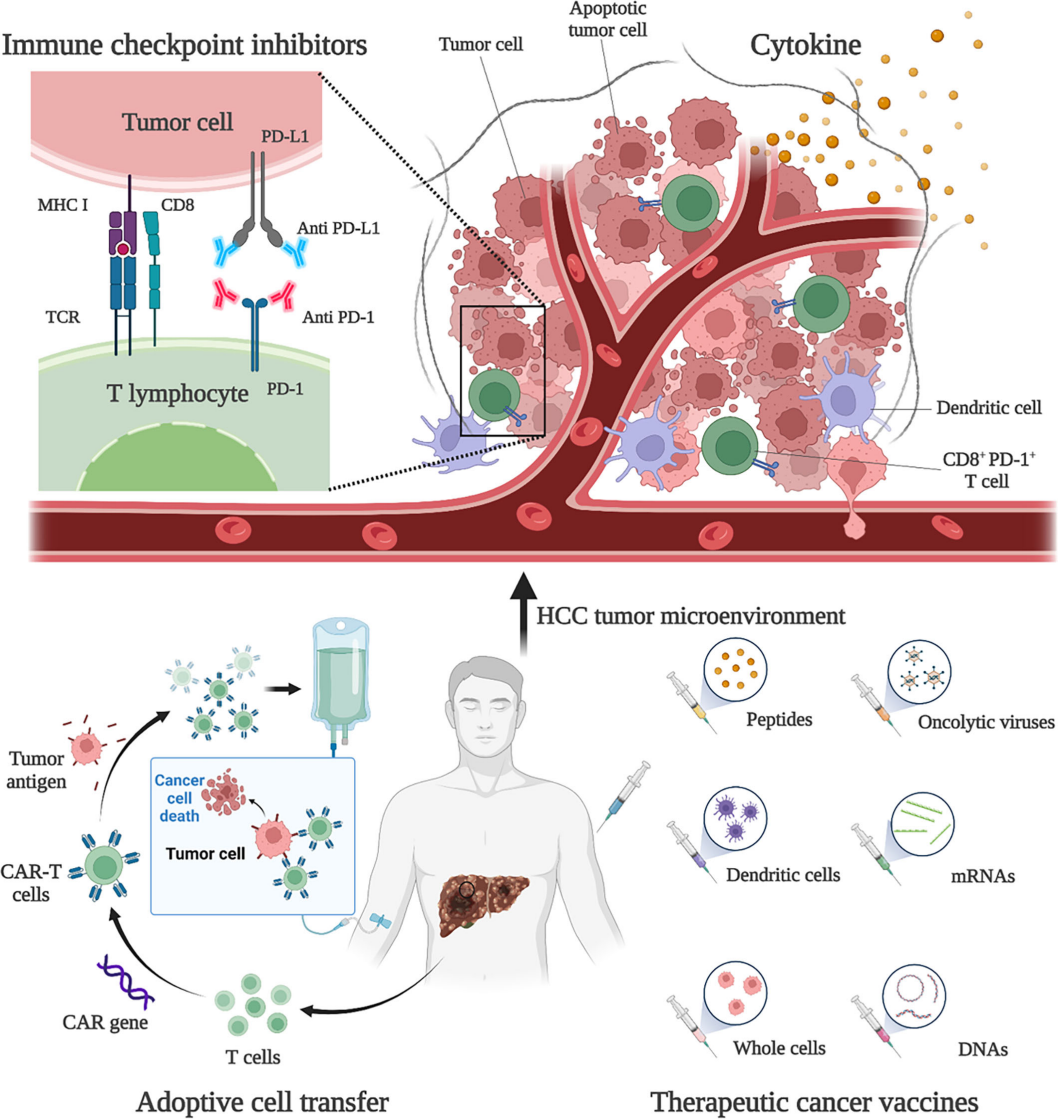
Immunotherapies for HCC. Current HCC immunotherapy strategies include immune checkpoint inhibitors (ICIs), cytokine-based therapies, adoptive cell transfer (ACT), and therapeutic vaccines. Anti-PD-1 and anti-PD-L1 treatments are examples of ICIs therapy. By blocking PD-1 and PD-L1, the anti-tumor activity of CD8^+^ T cells can be restored. An example of ACT therapy is CAR-T therapy. CAR-T therapy is derived from immune cells extracted from patients’ peripheral blood and genetically engineered to express chimeric antigen receptors (CARs). These CARs can recognize specific cancer antigens and stimulate the immune destruction of tumor cells. Therapeutic vaccines include peptides, DCs, whole-cell vaccines, oncolytic viruses, mRNAs, and DNA preparations to increase or achieve a specific immune response to tumor antigens.

HCC is usually developed from chronic liver disease, such as chronic hepatitis B, and is therefore considered to be inflammatory cancer. This inflammation promotes the transformation of liver cells and contributes to cancer (48). As inflammatory cancer, patients with high lymphocyte density in HCC tumors tend to have a better prognosis (6). Therefore, immunotherapies are considered as ideal treatment for HCC. Existing treatment options for HCC, such as surgery, adjuvant chemoradiotherapy, liver transplantation and radiofrequency ablation, do not benefit all patients, and a more comprehensive approach is needed. Immunotherapies have been shown to be effective and safe in the treatment of a large number of solid tumors (e.g., malignant melanoma and non-small cell lung cancer), extending the overall survival (OS) and providing tolerable toxicity, which are revolutionizing the management of cancer (49). Existing HCC immunotherapy strategies include ICIs, cytokine-based therapies, cell-based therapies, and tumor vaccines (**Figure 1**). However, due to the low tumor mutation load (TML) and the special immunosuppressive microenvironment, the application of HCC immunotherapies is facing challenges and further optimization strategies are needed (50).

#### 2.2.1 Immune checkpoint inhibitors

ICIs are monoclonal antibodies that block immune checkpoint molecules that inhibit the anti-tumor immune response. Immune checkpoint molecules are key modulators of anti-tumor T cell responses and can be expressed not only by T cells, but also by antigen-presenting cells (such as DC and macrophages) and tumor cells. Major inhibitory immune checkpoint receptors naturally inhibit T cell activity and play a critical role in maintaining self-tolerance, also mediating immune-escape of cancer cells (7). Currently, the targeted therapies of PD-1, its ligand PD-L1 and CTLA-4 have been fully studied and have become the pillar of immunotherapy for solid tumors (51).

The interaction between PD-L1 and PD-1 leads to widespread dephosphorylation of T-cell-activated kinases, resulting in T-cell inactivation. This effect mediates the immune tolerance of tumors (52). Studies have shown that PD-L1 is expressed in 82% of HCC samples, and the expression rate in HBV-positive patients is higher than that in HBV-negative patients (53). Therefore, blocking PD-1 or PD-L1 can restore the function of CD8^+^ T cells and exert anti-tumor function in HCC patients. Currently, the clinical value of PD-1 or PD-L1 inhibitors has been widely demonstrated and approved for use in several countries. Existing drugs include nivolumab, pembrolizumab and atezolizumab. Nivolumab is a human anti-PD-1 IgG4 monoclonal antibody that blocks PD-1 and was approved by the FDA in 2017 for second-line advanced HCC patients with sorafenib progression. Clinical studies have shown that nivolumab has a manageable safety profile and shows sustained antitumor activity in patients with advanced HCC (17). In a study of 743 HCC patients, first-line nivolumab and sorafenib-treated patients had comparable overall survival (15.2 *vs* 13.4 months) and showed a good safety profile (54). An Asian cohort study showed a response rate of 15% for nivolumab in HCC patients who had already been treated with sorafenib (55). Nivolumab combined with ipilimumab (an antibody against CTLA-4) showed better efficacy and safety in the treatment of advanced HCC patients. The objective response rate was 32% (95% CI, 20%-47%) in 148 subjects using a combination regimen (4 doses of nivolumab 1 mg/kg + ipilimumab 3 mg/kg every 3 weeks, then nivolumab 240 mg every 2 weeks) (56). In addition, atezolizumab, an IgG1 monoclonal antibody targeting PD-L1, and the anti-VEGFA antibody bevacizumab have produced better outcomes in advanced HCC patients than sorafenib and have become the new standard treatment for patients with unresectable HCC. Atezolizumab in combination with bevacizumab (AtezoBev) has been shown to be repeatable safe and effective in routine clinical practice (57). In a phase Ib trial, of 104 unresectable HCC patients treated with atezolizumab in combination with bevacizumab, 37 (36%; 95% CI 26%-46%) patients achieved a confirmed objective response (58). In a comparative trial, atezolizumab combined with bevacizumab showed better 12-month survival (67.2% *vs* 54.6%) and progression-free survival (6.8 *vs* 4.3 months) than sorafenib (59). Further molecular mechanism studies have also confirmed that anti-VEGF can act synergically with anti-PD-L1 to target angiogenesis, T_reg_ proliferation and myeloid cell inflammation (60). Pembrolizumab, an anti-PD-1 IgG4 monoclonal antibody, also demonstrated high efficacy and tolerability in patients with advanced HCC. In a Phase III study, pembrolizumab had a median OS of 13.9 months for advanced HCC (61). In 2020, atezolizumab plus bevacizumab became the standard first-line systemic therapy for advanced HCC, and the monotherapies pembrolizumab and nivolumab plus ipilimumab are currently approved as second-line therapy for patients with disease progression in first-line tyrosine kinase inhibitors (TKI) (49). It is important to note that some experimental and clinical studies of solid tumors seem to favor anti-PD-1 over anti-PD-L1 therapy. A meta-analysis of 19 randomized clinical trials showed that anti-PD-1 therapy resulted in better survival outcomes than anti-PD-L1 treatment (62). This finding may be partly attributed to the poor pharmacokinetic properties of anti-PD-L1 antibodies and the additional blocking effect of anti-PD-1 antibodies on PD-L2 (62).

CTLA-4 competitively inhibits the binding of the B7 ligand to the costimulatory receptor CD28, resulting in decreased peripheral T-cell activity. Inhibition of CTLA-4 can promote the increased activation of infantile CD4^+^ and CD8^+^ T cells, as well as the rebalancing of endogenous effector and regulatory regions in the TME (15). Anti-CTLA-4 treatment can activate and increase the abundance of CD4^+^ and CD8^+^ T cells, and reduce the clonability of peripheral T cells in HCC patients (63). Studies have shown that the expression of CTLA-4 in CD8^+^ and CD4^+^ T cells isolated from HCC tissues is significantly higher than that in tumor-free tissues or blood (64). Therefore, inhibition of CTLA-4 can play an antitumor role by enhancing T-cell activity in HCC patients. Ipilimumab, a CTLA-4 inhibitor, has been shown to be effective in combination with nivolumab for advanced HCC. This strategy has been approved in many countries for second-line advanced HCC patients with sorafenib progression (49). Tremelimumab is a fully human IgG2 monoclonal antibody that binds to CTLA-4 on the surface of activated T cells, thereby blocking its binding to CD28 (65). Current studies have proved that Tremelimumab combined with tumor ablation is feasible in the treatment of advanced HCC patients, with a partial response rate of 26.3%. This combination therapy resulted in the accumulation of CD8^+^ T cells in the tumor and decreased viral load in HCV patients (66). Although CTLA-4 inhibitors have achieved promising results in clinical trials to date, researches on the mechanisms of CTLA-4 blocking from HCC preclinical models are limited, and further studies are needed (15).

In addition to PD-1/PD-L1 and CTLA-4, blocking other co-inhibitory checkpoints such as LAG-3 or TIM-3 is also currently the focus of extensive clinical research. T_reg_ and CD8^+^ T cells isolated from HCC TME expressed more PD-1, LAG-3 and TIM-3 than those isolated from non-tumor microenvironment (NTME) by proteomics and transcriptomic analysis, and showed T cell inhibition (67). Lymphocyte activation gene 3 (LAG-3) is a membrane protein closely related to CD4. It is expressed by a variety of T cells, such as CD4^+^, CD8^+^, and T_reg_, as well as NK cells, DCs, and B cells. LAG-3 binds to MHC II of APC and prevents recognition of T cell receptors (TCRs), thereby inhibiting T-cell-mediated immune responses (50, 68). The density of LAG-3 positive cells increased significantly in HCC tumor tissues. Increased density of LAG-3^+^ cells and decreased level of CD8^+^ T cells were associated with poor prognosis (69). Studies have demonstrated the potential predictive and prognostic effects of LAG-3 as a serum biomarker in HCC patients undergoing transarterial chemoembolization (TACE) therapy. High LAG-3 levels before TACE are associated with poor disease outcomes and reduce overall survival (70). The expression of LAG-3 in tumor tissues is usually accompanied by an increased level of PD-L1 (71). Therefore, the development of LAG-3 inhibitors and their combination with anti-PD-1/PD-L1 may have significant synergistic clinical benefits. However, there are few clinical trials using these targets for HCC, and their efficacy has yet to be proven. T-cell immunoglobulin and mucin domain 3 (TIM-3) is an immunomodulatory receptor that binds to ligands on tumor cells and the microenvironment and inhibits antitumor immunity in a variety of cancers, including HCC. TIM-3 is one of the main inhibitory receptors on NK cells, which can mediate the reduction of anti-tumor ability (72, 73). At present, there are relatively few studies on how TIM-3 inhibits NK cells in HCC. This may be related to an endogenous ligand called phosphatidylserine (PtdSer). PtdSer is involved in promoting the phosphorylation of TIM-3, which then competes with PI3K p110 to bind p85 and inhibit the downstream Akt/mTORC1 signaling pathway, leading to NK cell dysfunction. Gene ablation, antibody-based functional blocking and lentivirus-mediated TIM-3 inhibition can inhibit HCC growth by restoring cytokine secretion and cytotoxicity of NK cells (74).

Overall, ICIs have several advantages over other types of immunotherapies, such as cell-based therapies, in terms of commercial availability, suitability, and not being limited by human leukocyte antigen (HLA) status. Although many trials showed promising results with ICIs in patients with advanced HCC, more trials are needed to show efficacy as a first-line treatment and in combination with other immunotoxic or cytotoxic therapies. Moreover, some new immune checkpoint inhibition therapeutic strategies need further mechanism studies and clinical validation (65).

#### 2.2.2 Vaccine therapy and cell-based therapy

Measurable T-cell responses to tumor-associated antigens expressed by HCC cells, such as AFP, GCP3, and MUC1, have guided the development of antigen-specific therapeutic vaccines and cell therapies (75). These strategies play a therapeutic role by activating or enhancing tumor immunity in HCC patients through the introduction of tumor antigens or tumor-associated antigen (TAA) sensitive immune cells *in vitro*. HCC vaccine therapy utilizes similar immune recognition principles and promotes an adaptive immune response to specific antigens. This method can not only be used for cancer prevention, but also for cancer treatment (49). Classical tumor vaccines involve exogenous antigens or antigen pulsed DCs. One strategy is to transfect DCs with a pulse of tumor cell lysate or with a TAA-expressing vector. Adoptive transfer of these modified DCs into patients was used to optimize the immunogenicity of secreted cancer antigens (including AFP) in response to weakened natural immune responses or functional abnormalities in HCC patients (76). Therapeutic vaccines include peptides, DCs, whole-cell vaccines, oncolytic viruses, mRNAs and DNA preparations to increase or achieve a specific immune response to tumor antigens (77). The key to vaccine therapy is that tumor antigens should provide sufficient immunogenicity to break the tolerance imposed by the many self-molecules expressed by tumor cells. At the same time, the antigen should confer specificity on tumor cells and avoid unnecessary recognition of non-tumor cells. So, screening for the right antigens is challenging. Due to the special immunosuppressive environment of HCC, it is also unknown whether antigen input can induce a strong enough immune response (78). Combination therapy and new vaccine synthesis strategies can overcome these challenges. For example, the combination of ICIs and tumor vaccine treatment can enhance the activity of T cells by blocking immunosuppressive factors, thus enhancing the function of the vaccine (79). The development of new tumor vaccines using nanotechnology has also contributed to the advancement of this therapeutic approach (80). For example, one study tried to use DC-derived exosomes (DEX) as a non-cellular vaccine for tumor immunotherapy. By anchoring HCC targeting peptide p47 (P) and an alpha-fetal protein epitope (AFP212-A2) to DEX, the researchers produced a novel vaccine, DEXP&A2&N. DEXP&A2&N achieves tumor-targeted delivery of high-mobility group nucleosome binding protein 1 (HMGN1; N1ND-N) and promotes N1ND-mediated endogenous DC recruitment and activation in tumors in the presence of HCC antigens. To achieve cross-presentation of tumor antigens and induce tumor-specific T-cell responses (81).

Another strategy for immune regulation of antitumor responses is adoptive cell transfer (ACT). ACT is a highly personalized form of cancer immunotherapy involving the metastasis of host-derived amplified immune cells (82). ACT therapy for HCC includes tumor infiltrating lymphocytes (TILs), cytokine induced killer cells (CIKs), and CAR-T (50). Adoptive metastasis of TIL has been shown to produce complete and lasting tumor regression in patients with metastatic melanoma, and its efficacy in HCC remains to be demonstrated (83). Another ACT strategy tried in adjuvant therapy for HCC is the use of CIK. CIK cells are autologous cells amplified *in vitro* from peripheral blood mononuclear cells of patients cultured with cytokine cocktails and anti-CD3 antibodies. CIK cells consist of a variety of subpopulations: CD3+/CD56+ cells, CD3−/CD56+ NK cells, and CD3+/CD56− cytotoxic T cells. Therefore, CIK cells have the dual function of T cells and NK cells, with a strong anti-tumor effect (84). Current studies have proved that CIK is an effective adjunctive therapy in early HCC. For advanced HCC, CIK can also show a good therapeutic effect by targeting MDSCs to reduce their immunosuppressive function (85). CAR-T therapy, as a new ACT, has made considerable progress in the treatment of HCC. CAR-T therapy takes immune cells from patients’ peripheral blood and genetically engineers them to express chimeric antigen receptors (CARs). These cell membrane proteins bind to specific cancer antigens and stimulate the immune destruction of tumor cells (86, 87). It was shown that CAR-T therapy inhibited tumors through multiple mechanisms. For example, a CAR-T therapy targeting Glypican 3 (GPC3) has been shown to be effective against HCC in mice. The mechanisms involved include inducing perforin and granulozyme-mediated apoptosis and reducing the level of active β-catenin in HCC cells. This is because GPC3 is a cancerous fetal antigen involved in Wnt-dependent cell proliferation (88).

#### 2.2.3 Cytokine-based therapy

For patients with HCC, cytokine-based therapies have met limited benefits. The use of interferon (IFN) seems to be a reasonable first choice for HCC treatment, which may have both antiviral and antitumor functions. It has been demonstrated that the combination of IFN-α and IL-24 can inhibit HCC by promoting tumor apoptosis and reducing angiogenesis (89). However, patients with cirrhosis and HCC have poor tolerance to IFN therapy, resulting in nearly half of the patients discontinuing treatment due to intolerance or adverse events (90).

### 2.3 Resistance to immunotherapies

Due to the microenvironmental specificity of the liver, the TME in HCC exhibits high immunosuppression and drug resistance, resulting in excessive or insufficient responses to immunotherapies (91). Recent studies have revealed the underlying mechanisms of immunotherapy resistance, which can be divided into primary resistance and adaptive or acquired resistance. Primary resistance is characterized by tumor failure to respond to immunotherapy, which may be due to T cells’ lack of tumor antigen recognition. When the patient’s immune system is able to recognize tumor antigens, the tumor can also protect itself from immune attack through adaptive or acquired resistance. The occurrence of drug resistance may be due to intrinsic characteristics of the tumor, such as low tumor mutation load and high PD-L1 expression, or extrinsic characteristics of the tumor, such as the absence of T cells with antigen-specific TCRs and high immunosuppressive TME (92). Specific to each type of immunotherapy, their resistance mechanisms are very complex and involve many factors. Take ICIs, for example. Although some ICIs (such as anti-PD-1 antibodies and anti-CTLA-4 antibodies) have been approved for first-line or second-line treatment of HCC in some countries, some advanced HCC patients do not respond to therapy, and the overall response rate remains low (93). This may be related to immune-regulatory metabolite production in HCC TME. In one HCC model, the use of ICIs led to an increase in IFN-γ-dependent expression of indoleamine 2, 3-dioxygenase (IDO) in tumor cells. Among them, an increase in tumor-derived IDO1 promotes resistance to ICIs therapy. The combination of IDO inhibitors can enhance the efficacy of ICIs (94). Another potential cause of ICIs resistance is the production of anti-drug antibodies (ADAs), which can alter the clearance of these drugs or neutralize their activity. It is not clear whether ADAs cause resistance to HCC. However, ADAs were detected in up to 36% of Non-small cell lung cancer (NSCLC) patients treated with atezolizumab, which has a negative impact on systemic exposure to the drug and has detrimental effects on anti-tumor efficacy (7). In addition to the effects of TME, genetic and epigenetic defects in patients themselves can induce immune evasion of tumor cells, further affecting the response to ICIs. For example, genetic and epigenetic aberrations that lead to defective antigen presentation can promote primary and acquired resistance to ICIs (95). Some studies have also demonstrated the role of signal-related mutations in tumor resistance to ICIs. For example, some mutations can activate the Wnt/β-catenin pathway, thus leading to changes in tumor PD-L1 and triggering the occurrence of ICIs resistance (96).

Although some resistance mechanisms have not been demonstrated in HCC, further optimization of HCC immunotherapy strategies is imperative. New directions have been opened for the development of immunotherapy by combining different treatments or by using new technologies to synthesize new immunotherapy drugs. Recent studies have shown the value of combination immunotherapy and epigenetic therapy. Among them, HDACis combined with immunotherapy has achieved better results in HCC treatment. Moreover, nano-based drug delivery systems (NNDS) built using nanotechnology further optimize existing treatment options.

## 3 HDACs and HDACis treatment for HCC

### 3.1 Histone deacetylation modification and HDAC inhibitors

The nucleosome is the basic unit of chromatin and is made up of DNA and histones. Histones are a group of small, positively charged proteins that include H1, H2A, H2B, H3 and H4. Histones are essential in packaging DNA into cells, chromatin and chromosomes. The histone core octamer is composed of H2A, H2B, H3, and H4. They are wrapped in a 147-base pair DNA band and linked by H1 (97). Covalent modifications of histones are central to the regulation of chromatin dynamics which comprise methylation, phosphorylation, acetylation, ubiquitylation, sumoylation, glycosylation, and ADP-ribosylation (98). Many biological processes involving chromatin, such as transcription, DNA repair, replication, and genome stability, are regulated by chromatin and its modifications. N^ϵ^-acetylation of lysine residues is a major histone modification involved in transcription, chromatin structure and DNA repair. Acetylation neutralizes the positive charge of lysine and weakens the electrostatic interaction between histones and negatively charged DNA. Thus, histone acetylation is often associated with a more “open” chromatin conformation (99). Acetylation is highly dynamic and regulated by the competitive activity of two enzyme families, histone acetyltransferases (HATs) and histone deacetylases (HDACs). These two enzymes alter the state of chromatin, which in turn affects gene transcription and genome stability. Abnormalities in the functioning of these two enzymes have also been shown to play a role in the development of cancer. In contrast to DNA mutations, epigenetic changes represent reversible changes that offer the possibility of truly “restorative” therapeutic interventions. Great progress has been made in the therapeutic strategies targeting HDACs (100).

HDACs reverse lysine acetylation and restore positive the charge on the side chain, causing chromatin to contract. HDACs consist of 18 enzymes from two families and can be divided into 4 groups based on their sequence homology and domain organization. Class I HDACs (HDAC-1, HDAC-2, HDAC-3, HDAC-8) are located in the nucleus, widely expressed in various tissues and involved in gene expression. Class II HDACs are divided into two subgroups, Class IIa (HDAC-4, HDAC-5, HDAC-7, and HDAC-9) and Class IIb (HDAC-6 and HDAC-10), which are involved in cell differentiation. Class IIa HDACs shuttle between cytoplasm and nucleus. Class IIb HDACs are located in the cytoplasm. Class I HDACs and Class II HDACs represent the HDACs most closely associated with yeast *sc*Rpd3 and *sc*Hda1, respectively. Class IV HDACs include only one enzyme, HDAC-11. Class I, II, and IV HDACs share related catalytic mechanisms that require Zn^+^ but do not involve the use of cofactors. In contrast, Class III HDACs (sirtuin 1-7) are homologous to yeast *sc*Sir2 and employ a unique NAD^+^-dependent catalytic mechanism (99, 101, 102). In addition, HDACs are known to regulate a variety of non-histone targets, such as tubulin, heat shock protein 90 (HSP-90), and *p53*, thereby affecting cell growth, apoptosis, invasion, and angiogenesis (103). It has been found that HDAC-6 is involved in α-tubulin deacetylation, affecting mitosis and other processes dependent on microtubule network acetylation patterns (104).

Abnormal HDACs are involved in the occurrence and development of many tumors, including cell proliferation, cell migration, cell death, and angiogenesis. HDACs shrink chromatin through deacetylation, resulting in transcriptional silencing of tumor suppressor and apoptosis genes, disrupting the balance between oncogenes and oncosuppressor genes. Many non-histone transcription factors, such as HSP-90 and tubulin, are also substrates for HDACs (101). Chimeric fusion proteins in leukemia, such as PML-RARα, PLZF-RARα, and AML1-ETO, have been shown to recruit HDACs to mediate abnormal gene silencing, which contributes to the development of leukemia (105). HDACs have also been found to be overexpressed or overactive in various solid tumors and inhibit the expression of tumor suppressor genes, leading to uncontrolled proliferation and inhibiting cell repair and apoptosis (102). Studies have shown that HDAC-5 can directly interact with T-box3 (a transcriptional suppressor) to jointly inhibit the expression of E-cadherin and promote the metastasis of tumor cells (106). Therefore, HDACs may be promising drug targets for cancer treatment. Currently, HDACis have been shown to be powerful in the treatment of cancer (107, 108).

HDACi reverses some abnormal gene inhibition in malignant tumors and induces growth arrest, differentiation, and apoptosis of cancer cells (**Table 1**). There are currently four HDACis approved by the FDA for cancers (99). The first approved HDACi is suberoylanilide hydroxamic acid (SAHA) or vorinostat for the treatment of refractory CTCL. The second is romidepsin for CTCL and peripheral T-cell lymphoma (PTCL). The third drug approved as an HDACi was panobinostat for oral use, in combination with bortezomib and dexamethasone for the treatment of relapsed multiple myeloma. The fourth, belinostat, is used for the treatment of PTCL. In addition, another HDACi chidamide was approved in China for the treatment of hematologic malignancies (101, 109). These drugs have produced impressive clinical data. In a Phase II trial, chidamide showed significant single-agent activity and controlled toxicity in relapsed or refractory PTCL. 79 patients with PTCL histology who received chidamide had an overall survival of 21.4 months. Patients with vascular immunoblastic T-cell lymphoma (AITL) had a higher ORR (50%) and a 40% complete response/unconfirmed complete response (CR/CRu) on chidamide, as well as a more durable response (110). Microarray experiments show that < 10% of the genome showed significant changes in expression after HDACis treatment. In cancer cells, these perturbations appear to disrupt their metastases and lead cells to non-proliferative destinies, including differentiation, immune regulation, chromatin instability, reduced DNA damage repair, reactive oxygen species production, cell cycle arrest, apoptosis, autophagy, and reduced angiogenesis and cell migration. For example, HDACis can restore *p53* protein transcription and thus induce apoptosis of drug-resistant cancer cells (30, 109, 111). Some of the newer HDACis are now being shown to work in a wide range of tumors. For example, one study demonstrated the ability of a modified novel highly selective HDAC I/IIb inhibitor, Purinostat Mesylate (PMF), to treat chronic myelogenous leukemia (CML). PMF can significantly prevent the progression of BCR-ABL(T315I) induced CML by inhibiting leukemia stem cells (LSCs). This may provide a new treatment strategy for TKI-resistant CML patients in the future (112). Thailandepsin A (TDP-A) is another novel HDACi with extensive anti-proliferative activity. It has been proved that TDP-A can inhibit proliferation and induce apoptosis of breast cancer cells at low nanomolar concentrations. Furthermore, TDP-A has strong selective inhibition on Class I HDACs, such as HDAC-1, HDAC-2 and HDAC-3, and weak inhibitory activity on HDAC-4 and HDAC-8. This selectivity makes TDP-A a promising epigenetic drug for cancer treatment (113).

**Table 1:** HDAC inhibitors for tumors.

HDAC inhibitors	HDAC specificity	Tumors
Vorinostat	Class I, II, IV	CTCL
Romidepsin	Class I	CTCL, PTCL
Panobinostat	Class I, II, IV	MM
Belinostat	Class I, II, IV	PTCL, HCC
Chidamide	Class I, IIb	Hematologic malignancy
Trichostatin A	Class I, II, IV	Broad cancers
Givinostat	Class I, II, IV	Leukemia
Entinostat	Class I	Hematologic malignancy, breast cancer
Mocetinostat	Class I, IV (HDAC-1, HDAC-2, HDAC-3, HDAC-11)	Hematologic malignancy, lung cancer
Rocilinostat	Class IIb (HDAC-6)	MM, lymphoma, lung cancer, breast cancer
Nicotinamide	Class III (SIRT-3)	Skin cancer
Cambinol	Class III (SIRT-1, SIRT-2)	Lymphoma, breast cancer
Quisinostat	Class I, II, IV (HDAC-1, HDAC-2, HDAC-4, HDAC-10, HDAC-11)	MM, multiple solid tumors
Purinostat Mesylate	Class I, IIb	CML
Thailandepsin A	Class I (HDAC-1, HDAC-2, HDAC-3)	Breast cancer

HDAC, histone deacetylase; SIRT, sirtuin; CTCL, cutaneous T-cell lymphoma; PTCL, peripheral T cell lymphoma; HCC, Hepatocellular carcinoma; MM, multiple myeloma; CML, Chronic myelogenous leukemia.

However, HDACis also face some challenges in treating cancer. One concern is the multipotency of drugs and their targets. Currently used HDACis are mostly non-selective pan-HDACis, whose relatively low specificity may alter the expression of thousands of important genes, leading to adverse consequences and hindering the wide clinical application of HDACis (99, 114). It is also challenging to determine the dosage of HDACis. Doctors need to find a treatment window that allows higher doses to be administered to more aggressive cancers, taking into account patients’ tolerance (105). HDACis resistance is another challenge. Studies have shown that tumor cells can develop resistance through compensatory changes in HAT/HDAC expression levels, induction of p21 and thioredoxin, and drug effluence by ATP-binding cassette transporters (109). Combining HDACis with other drugs is a credible way to address these challenges. However, combination therapy still faces many problems, such as different drug solubility, resulting in physical incompatibility, which leads to formula precipitation or drug inactivation, requiring reformulation. In addition, there is an increased risk of drug-drug interactions and an increased tendency for adverse reactions (101). Chimeric HDACis synthesized by molecular hybridization (MH) strategy is a new development direction of HDACis. By combining drugs with different therapeutic effects, such as TKI and HDACi, in a single molecule, new drugs with better affinity and efficacy can be created. A highly effective dual inhibitor targeting bromodomain and extra-terminal (BET) and HDACs for pancreatic cancer has been reported. The antitumor activity of this dual inhibitor was higher *in vivo* and *in vitro* than that of BET inhibitor and HDACis alone or in combination (115).

### 3.2 HDAC inhibitors for HCC treatment

The dysregulation of HDACs and their roles in HCC development are being actively studied (**Table 2**). At present, there have been many reports indicating that HDACs are over-expressed or over-activated in HCC patients. Some of these studies have demonstrated the relationship between the overexpression of Class I HDACs such as HDAC-1 and HDAC-2 in HCC tissues and the increased mortality and poor prognosis of patients (124). Currently, many molecular classifications and prognostic gene markers for HCC patients have been established based on genome-wide gene expression profiles. A recent study systematically assessed the effect of these genetic characteristics on prognosis and identified valuable prognostic biomarkers by integrating these genetic characteristics. Tissue microarray analysis of 60 HCC patients showed that the expression level of HDAC-2 was negatively correlated with OS in HCC patients. The expression level of HDAC-2 in tumor tissues is significantly higher than that in adjacent normal tissues, and is associated with poor survival in HCC patients (125). Class II and III HDACs, such as HDAC-4, HDAC-5, SIRT-1, SIRT-2, and SIRT-7, have also been found to be up-regulated in HCC, and their correlation with tumor progression has been demonstrated in some cases (126). A large number of mechanism studies have shown that HDACs are involved in the pathogenesis of HCC. When overexpressed, these epigenetic modification factors exhibit various cancer-promoting effects, including inhibiting the expression of tumor suppressor genes, activating cell cycle progression, escaping apoptosis, adapting to hypoxia, and metabolic reprogramming. The interactions between HDACs and other carcinogenic molecules are also quite complex (125). In contrast, some HDACs appear to play a role in tumor inhibition in HCC. For example, HDAC-6 is a unique tumor suppressor in HCC. Inhibition or inactivation of HDAC-6 can promote the development of the tumor (127). The discovery of these mechanisms not only explains the role of HDACs in HCC, but also provides targets for targeted therapy. For example, existing studies have demonstrated that HDAC-2 is associated with poor prognosis in HCC, suggesting that inhibiting HDAC-2 may be a potential strategy to improve prognosis in HCC patients. In fact, in HCC cells, inhibition of HDAC-2 disrupts the G1/S phase of the cell cycle and ultimately leads to apoptosis by upregulating total *p21*, *p27* and acetylated *p53* and reducing the expression levels of some oncogenes (125, 128). These results are consistent with the above conjecture.

**Table 2:** HDAC inhibitors for HCC treatment.

Treatment strategies	HDAC specificity	Mechanisms	References
Trichostatin A	Class I, II, IV	• Decrease the expression of oncogene *c-Met* and increase the level of MicroRNA-449	(116)
Trichostatine A + curcumin	Class I, II, IV	• Inhibition of NF-κB signaling pathway • sensitize resistant tumor cells to the curcumin treatment.	(117)
Belinostat	Class I, II, IV	• Inhibit histone deacetylase and reverse the up-regulation of oncogenes	(118)
Resminostat + sorafenib	Class I, IIb (HDAC-1, HDAC-3, HDAC-6, HDAC-8)	• Inhibition of histone acetylation associated with sensitivity and tolerance to sorafenib	(119)
TMP269 + lenvatinib	Class IIa	• Down-regulate FGFR4 and block FGFR signaling in FGFR4-positive HCC cell lines	(120)
Panobinostat + radiotherapy	Class I, II, IV	• Inhibit nuclear translocation and dissociate the HDAC4/Ubc9/Rad51 complex to impair DNA repair	(121)
AR42 + telomerase-specific oncolytic adenoviral therapy	Class I, II, IV	• Decrease telomerase-induced phosphorylated Akt activation and enhance telomerase-induced apoptosis	(122)
SAHA+ FOXO1 inhibitor AS1842856	Class I, II, IV	• Inhibition of autophagy mediated by AMPK-FOXO1-ULK1 signaling axis • Preventing EMT induced cancer cells metastasis	(123)

HDAC, histone deacetylase; FGFR, fibroblast growth factor receptor 4; Ubc9, ubiquitin-conjugating enzyme 9; SAHA, suberoylanilide hydroxamic acid, vorinostat; AMPK, AMP-activated protein kinase; FOXO1, forkhead box o1; ULK1, Unc-51-like kinase 1; EMT, epithelial-mesenchymal transition.

The role of HDACs in the development of HCC is related to the regulation of acetylation of oncogenes (e.g., *c-Met* and *c-Myc*) and oncosuppressor genes (e.g., *p53*). Trichostatin A has previously been shown to effectively inhibit *c-Met* expression and promote apoptosis of HCC tumor cells (116). A recent study found that HDAC-3 and tumor necrosis factor receptor-associated factor 6 (TRAF6), an E3 ubiquitin ligase, are jointly involved in significant upregulation of the oncogene *c-Myc* in HCC, thereby promoting malignant transformation and progression of tumors. TRAF6 disrupts the binding of HDAC-3 and *c-Myc* promoters, resulting in histone acetylation and epigenetic enhancement of *c-Myc* mRNA expression. This process also ultimately leads to increased stability of the *c-Myc* protein (129). In addition, a long non-coding RNA (lncRNA) that can be trans-activated by the *p53* gene, lnc-Ip53, can block *p53* acetylation by inhibiting the degradation of HDAC-1. This mechanism can lead to the loss of *p53* activity and the subsequent generation of tumor cell proliferation and apoptosis resistance (130).

Recent studies have also indicated the effects of HDACs on HCC cancer stem cells (CSCs), including maintaining cancer cell dryness and promoting self-renewal and proliferation. CSCs can cause tumor recurrence and metastasis, and play an important role in the generation of multi-drug resistant cancers (131). The promotion of HDACs on CSCs is achieved by affecting multiple signaling pathways. Recent studies have pointed to the key role of HDAC-11 in maintaining the dryness of HCC CSCs, while inhibition of HDAC-11 can promote apoptosis of cancer cells. HDAC-11 overexpression also reduced the sensitivity of HCC to sorafenib. This may be related to the regulation of HDAC-11 on the enhancement of glycolysis of HCC CSCs. CSCs require glycolysis and lipid metabolism for energy, and give priority to glycolysis for homeostasis (132). Further studies have shown that knockout of HDAC-11 in mice can promote histone acetylation of liver kinase B1 (LKB1) promoter region to increase LKB1 transcription, thus activating adenosine 5’-monophosphate (AMP)-activated protein kinase (AMPK) signaling pathway and inhibiting glycolysis pathway, thus inhibiting cancer dryness and HCC progression (133). In addition, HDAC-2 also promotes the proliferation and renewal of HCC CSCs by activating the Hedgehog (Hh) pathway. In this process, HDAC-2 and lnHDAC -2 (a lncRNA highly expressed in HCC and related to HDAC-2) co-inhibit the expression of patched 1 (PTCH1), thus activating Hedgehog signaling pathway and maintenance of hepatic CSCs dryness (134). The discovery of the mechanism of HDACs on CSCs provides a new target for combination therapy to overcome drug resistance in HCC tumors. For example, a recent study found that combined with Class I/II HDACis trichostatine can effectively improve the efficacy of inhibitor of kappa B kinase (IKK) in the treatment of drug-resistant HCC. Curcumin inhibits class I and II HDACs by inhibiting the NF-kB signaling pathway, which is enhanced by trichostatine combination therapy, sensitizing resistant tumor cells to curcumin therapy (117).

Currently, the mechanism of HDACis monotherapy for HCC is still in the stage of exploration, and its clinical effect remains to be proved. A Phase II trial previously demonstrated tolerable cytotoxicity of belinostat in HCC (118). Further pharmacokinetic studies demonstrated that belinostat was mainly metabolized through the glucoaldehyde pathway (135). In addition, HDACis have demonstrated excellent adjunctive therapeutic capabilities to enhance the efficacy of multiple HCC therapies. In terms of chemotherapy, HDACis have shown better adjuvant effect in many studies. HDACis can improve the efficacy of some traditional chemotherapy drugs (e.g., Fluoropyrimidines) against HCC and overcome resistance by targeting specific genes or proteins (35, 136). A phase I/II trial validated the combination of resminostat and sorafenib in the treatment of HCC. The results showed better safety and early signs of efficacy (119). Additionally, a new study has demonstrated that a selective class IIa HDACi (TMP269) enhances the efficacy of lenvatinib in fibroblast growth factor receptor 4 (FGFR4) positive HCC in mice (120, 135). Notably, the synergistic effect of HDACis allows these chemotherapeutic agents to exert their antitumor power without the need to reach very high doses. This effect greatly reduces the cytotoxicity of chemotherapy drugs, enabling them to be more widely used in the treatment of HCC, bringing a new development direction for the development of traditional drugs (35). For radiotherapy, it has shown that the use of HDAC-4 inhibitors can effectively enhance the killing efficiency of radiation on HCC tumor cells. Interruption of the HDAC-4 signaling pathway enhanced the radiation-induced mortality of cancer cells (121). In addition to traditional treatments, HDACis and several new treatments have shown good synergies. For example, Lin et al. demonstrated a synergistic therapeutic effect of pan-HDACi AR42 and telomerase-specific oncolytic adenovirus therapy. AR42 significantly enhanced telomerase-induced apoptosis in HCC tumor cells (122). These studies all showed the strong potential of HDACis in the field of HCC therapy.

Combination therapy can also overcome the occurrence of HDACi resistance and reduce the risk of drug use. Recently, studies have demonstrated that HDACis therapy can promote the epithelial-mesenchymal transition (EMT) of HCC through autophagy mediated by the AMPK-FOXO1-ULK1 signaling axis (123). EMT is a key step in tumor invasion and metastasis (137). This mechanism increases the risk of HDACis therapy, which leads to a limited therapeutic role in epithelial cell-derived cancers, including HCC. The combination of HDACis and FOXO1 inhibitors can effectively reduce this risk and increase the efficacy of treatment (123). Another example of combination therapy for HCC is the use of HDACis to enhance the efficacy of immunotherapy, which will be discussed in detail below. In general, HDACs play an important role in the development of HCC and provide new targets for more accurate treatment. HDACis have shown great value in the treatment and adjuvant therapy of HCC.

## 4 HDACis enhance the efficacy of HCC immunotherapy

### 4.1 HDACis enhance tumor immunotherapy

Immunotherapy has been successfully used in preclinical models or clinical settings to treat a variety of tumors, including HCC. However, the emergence of immunotherapy resistance is currently a major challenge. Although immunotherapy, such as immune checkpoint suppression therapy, has shown impressive clinical results, only some patients have achieved a lasting response (92). To overcome this problem, combination therapy strategies have been sought to achieve better efficacy. One strategy is to combine immunotherapy with HDACis. HDACis can potentially increase tumor immunogenicity, promote anti-tumor immune responses, or reverse immunosuppressive TME. Recently, HDACis combined immunotherapy has attracted much attention in cancer treatment (32) (**Table 3**).

**Table 3:** HDACi enhances the efficacy of immunotherapy.

HDAC inhibitors	HDAC specificity	Mechanisms	References
Vorinostat	Class I, II, IV	• Increase immunogenicity of tumor cells by increasing the expression of PD-1/PD-L1	(33)
Panobinostat	Class I, II, IV		(33)
OKI-179	Class I (HDAC-3)		(138)
Chidamide	Class I, IIb		(139)
Zabadinostat (CXD101)	Class I (HDAC-1, HDAC-2, HDAC3)	• Increase immunogenicity of tumor cells by increasing the expression of MHC molecule	(140)
Romidepsin	Class I	• Modulate the tumor microenvironment by increasing chemokine expression • Enhance the expression of NKG2D ligands and enhance the tumor killing ability	(141, 142)
PCI-34051 (for HCC)	Class I (HDAC-8)	• Reactivate the production of T-cell chemokines • Increase tumor infiltrating CD8^+^ T cells and enhance anti-PD-L1 therapy	(143)
HDAC-10 inhibitor (for HCC)	Class IIb (HDAC-10)	• Modulate the tumor microenvironment by increasing chemokine (CXCL10) expression	(144)
Tubacin (for HCC)	Class IIb (HDAC-6)	• Increase IL-17A in the tumor microenvironment • Increasing the expression of PD-1	(145)
Sodium valproate	Class I (HDAC-1, HDAC-2)	• Increase the expression of NKG2D ligand MICB by down-regulating miR-889	(146)

HDAC, histone deacetylase; PD-1/PD-L1, programmed death receptor-1/programmed death receptor-ligand 1; MHC, major histocompatibility complex; NKG2D, natural killer group 2D; CXCL10, C-X-C motif chemokine 10; IL-17A, interleukin-17A; MICB, major histocompatibility complex class I chain-related gene B; miR-889, microRNA 889.

HDACis increase the expression of PD-L1 and other immune checkpoints in tumor cells, which is an important mechanism to enhance the immunogenicity of tumor cells, and can improve the applicability and efficacy of ICIs. Abnormal expression of PD-L1 observed on the surface of human cancer cells mediates the inactivation of anti-tumor T cells and tumor immune escape (147). This mechanism also provides a target for PD-1/PD-L1 blockers to treat tumors. Studies have shown that the high expression of PD-L1 in tumors is one of the biomarkers to improve the sensitivity to PD-1/PD-L1 block (148). HDACis have been shown to increase PD-L1 expression in several tumors, including breast cancer, melanoma, HCC, soft tissue sarcoma, and B-cell lymphoma, thereby improving the efficacy of immunotherapy (33, 149, 150). Pan-HDACis, such as vorinostat and panobinostat, induce PD-L1 expression in B-cell lymphoma (33). Selective HDAC-3 inhibitors have also been shown to up-regulate the expression of PD-L1 in B-cell lymphoma, suggesting that HDAC-3 may be one of the key inhibitors mediating PD-L1 transcription in B-cell lymphoma (138). A similar mechanism has been found in soft tissue sarcomas (STS). Recent studies have shown that class I HDACi chidamide can increase histone acetylation of PD-L1 gene promoter in STS cancer cells and stimulate PD-L1 expression through activation of transcription factor STAT1. Further studies also demonstrated better efficacy of chidamide in combination with the anti-PD-1 antibody toripalimab in patients with advanced and metastatic sarcoma. Combination therapy also reduced the number of MDSCs in the TME, a key immunosuppressive cell population that mediates resistance to ICIs (139). HDACis’ increased PD-L1 expression and increased PD-1 blocking efficiency may be related to more drug therapeutic targets. Recently, it has been found in breast cancer that the highly expressed membrane PD-L1 can translocate into the nucleus mediated by HDAC-2, thereby regulating tumor gene expression. The effects of this mechanism are multiple (151). On the one hand, nuclear PD-L1 can regulate the expression of pro-inflammatory and immune response-related genes, promoting immune inflammation in the local TME and thus making tumors more sensitive to immunotherapy. On the other hand, this gene regulation also promotes distant metastasis of cancer and enhances tumor aggressiveness. In addition, nuclear PD-L1 also triggers the expression of other immune checkpoint molecules, leading to possible acquired immunotherapy resistance. Blocking nuclear translocation of PD-L1 using HDAC-2 inhibitors can reduce transcription of these immune checkpoint genes, leading to increased infiltration of CD8^+^ T cells and decreased levels of TNF-α in tumors (151, 152).

HDACis have also been shown to enhance tumor immunogenicity by promoting tumor antigen processing and presentation. The expression of MHC I in cancer is usually decreased due to epigenetic mechanisms, and HDACis can up-regulate the expression of MHC I in various types of cancer (34). Histone deacetylation usually induces chromatin shutdown of MHC II promoters, leading to MHC II downregulation in tumors, and HDACis can reverse this process (153). The effect of HDACis on tumor antigen processing and presentation enhances immunotherapy efficacy. Recently, a Class I HDACi CXD101 with selective activity was shown to enhance the efficacy of anti-PD-1 ICI in colorectal cancer. CXD101 induces the expression of molecules associated with antigen presentation, including MHC I, which increases antigen presentation and helps improve cytotoxic T cell conjugation and tumor cell killing. Anti-PD-1 antibodies release T cells by inhibiting immune checkpoints, which can then bind to MHC I with increased expression levels on tumor cells *via* T cell receptors, leading to increased cytotoxicity and tumor cell killing levels (140).

The regulation of the TME by HDACis is another important mechanism that enhances the efficacy of antitumor immunotherapy. Insufficient infiltration or abnormal function of anti-tumor immune cells, such as T cells and NK cells, is an important mechanism that causes tumor immune escape. HDACis can overcome this mechanism by recruiting more T and NK cells and enhancing their antitumor activity. HDACis enhance tumor immunogenicity essentially by activating more T cells to enhance the immune system’s ability to recognize and kill tumor cells. In addition, HDACi can also play an immune-enhancing role by inducing chemokine production and regulating the expression of activation or apoptosis-related ligands. Increased expression of T cell chemokines (e.g. CXCL10, CXCL10 and CCL5) in tumors is associated with better response to immunotherapy and improved patient outcomes (154, 155). In a mouse model, HDACi romidepsin significantly increased CXCL10 expression in lung cancer and induced a strong T-cell-dependent antitumor response (141). HDACis can induce tumor regression or rejection in various lung tumor models by promoting T cell recruitment and enhancing T cell function in combination with anti-PD-1 therapy. However, treatment with HDACis alone can lead to the overexpression of PD-L1 in tumor cells and the restriction of T cell function (150). Combined anti-PD-1 therapy can overcome this limitation by releasing IFN-γ and increasing the sensitivity of tumor cells to immunotherapy (141). This suggests that HDACis combined immunotherapy is a mutually reinforcing process that ultimately leads to a stronger synergistic therapeutic effect.

In addition to anti-tumor T cells, NK cells are also important immune components in the fight against tumors. HDACis can significantly enhance the expression of natural killer group 2D (NKG2D) ligands and activate NKG2D expressed in NK cells, thus enhancing the killing function of NK cells on tumors (142). Mechanistically, HDACis may enhance the expression of major histocompatibility complex class I-related chain A and B (MICA and MICB) and UL16 binding protein (ULBp) in tumor cells, which are key NKG2D ligands (156–158). This mechanism activates endogenous NK cells and enhances the toxicity of chimeric antigen receptor NK cell therapy (CAR-NK) to tumor cells. CAR-NK therapy refers to adding a chimeric antibody that can recognize tumor antigens and activate NK cells at the same time to enhance the anti-tumor ability of NK cells through genetic engineering (159). CAR-NK cells possess the dual intrinsic ability of natural receptors to recognize and target tumor cells. HDACis can increase the expression of NKG2D ligands to enhance the ability of CAR-NK cells to recognize tumors through natural receptors (32). Although many studies have demonstrated that HDACis enhance the anti-tumor response of NK cells by upregulating NKG2D, the results of a recent study suggest that HDACis may down-regulate another activating ligand, B7-H6, thereby inhibiting NK cell-mediated tumor cell recognition (160). This finding was confirmed in primary lymphoma and HCC samples and was associated with the inhibition of HDAC-3 (160). This suggests that combining HDACis with immunotherapy requires a rational strategy design, and that using non-selective HDACis may lead to unpredictable outcomes.

### 4.2 HDAC is combined with immunotherapy for HCC

**Figure 2:**
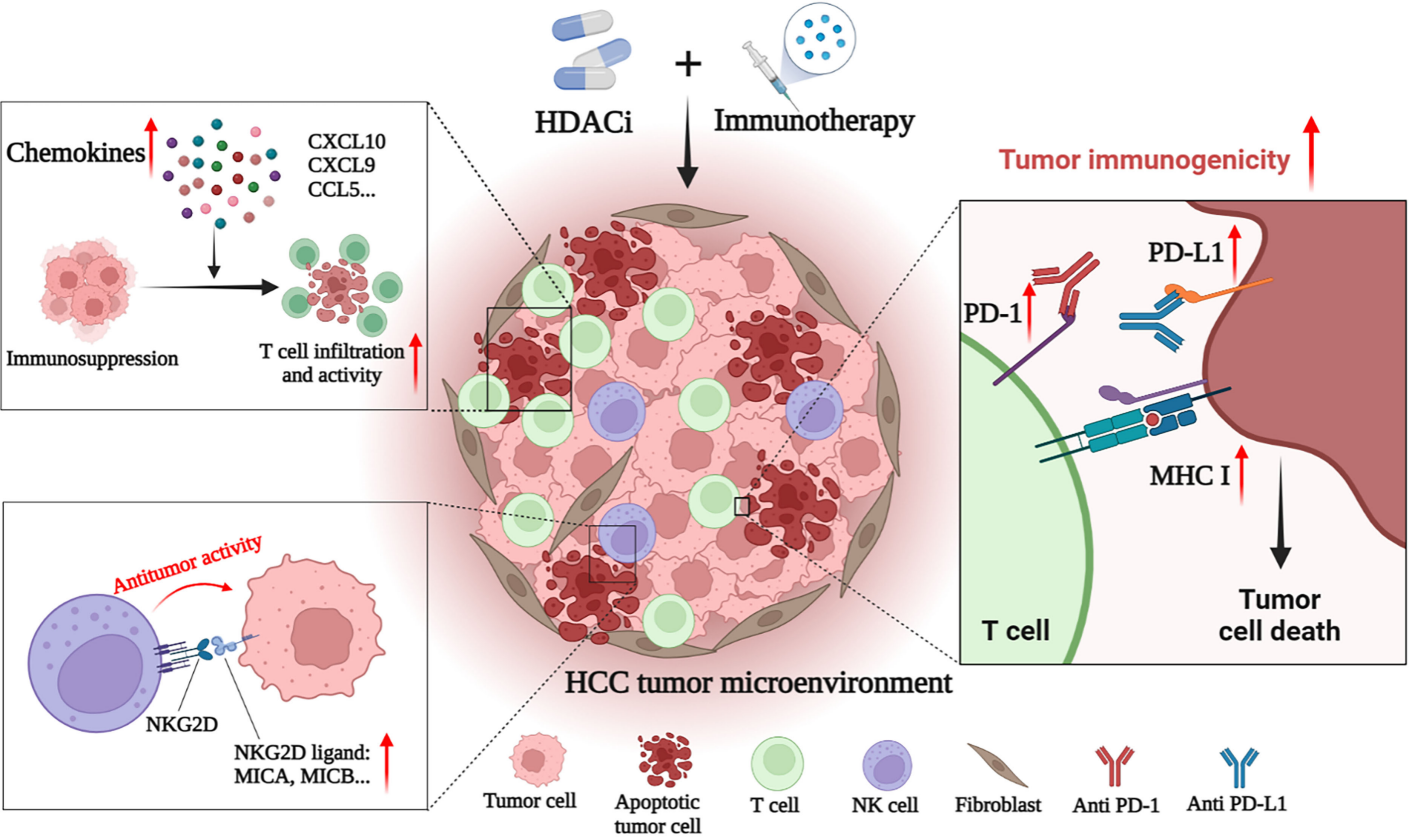
HDACi enhances the anti-tumor effect of immunotherapies in HCC. HDACi can enhance the efficacy of immunotherapy by enhancing the immunogenicity of tumor cells and modulating the tumor immune microenvironment. HDACi can increase the expression of PD-1 and PD-L1, thereby enhancing the sensitivity of HCC to ICIs. HDACi also facilitates the processing and presentation of tumor antigens by increasing the expression of MHC I and MHC II. HDACi can induce the expression of chemokines (CXCL10, CXCL9 and CCL5) in the microenvironment, increase the infiltration of T cells and enhance their antitumor activity. In addition, HDACi can increase the expression of NKG2D ligands in tumor cells, and activate and enhance the anti-tumor activity of NK cells.

HCC is a tumor with insufficient T-cell infiltration, which limits the effectiveness of immunotherapy in some patients (143). Therefore, additional mechanisms are needed to overcome HCC-induced immune tolerance and enhance the effectiveness of existing immunotherapy strategies. At present, many studies have shown that the combination of immunotherapies (such as ICIs) and HDACis may have a better therapeutic effect on HCC. For example, Belinostat has recently been shown to improve the efficacy of anti-CTLA-4 monotherapy and anti-CTLA-4 combined with anti-PD-1/PD-L1 in HCC patients, leading to complete tumor rejection (161). Mechanically, HDACis also improves the efficacy of immunotherapy by enhancing the immunogenicity of HCC cancer cells and regulating the TME. Specific mechanisms include up-regulation of PD-L1 expression, induction of chemokines, recruitment of T cells and NK cells, and enhancement of the anti-tumor function of immune cells (**Figure 2**) (143–146).

Zeste homolog 2 enhancer (EZH2) inhibition is one of the important mechanisms of HDACis enhancing HCC immunotherapy. EZH2, a histone H3 lysine methyltransferase, is a proven oncogene (162). HDAC-10 can induce EZH2 recruitment at the CXCL10 promoter of the chemokine and ultimately inhibit CXCL10 transcription (144). HDAC-10 is necessary for this process to occur and provides a target for treatment. By inhibiting the recruitment of EZH2 on the CXCL10 promoter, knockdown of HDAC-10 can promote the increase of CXCL10 expression in HCC, inducing the recruitment of T cells and NK cells, and regulating and controlling the anti-tumor response in the TME (144). In addition to inhibiting the expression of CXCL10, EZH2 has also been shown to inhibit the expression of PD-L1 and reduce the effect of anti-CTLA-4 therapy (163, 164). HDAC-8 inhibition has also recently been shown to promote chemokine production. Down-regulation of HDAC-8 increases global acetylation and enhancer acetylation of histone H3 lysine 27 (H3K27), thereby reactivating the production of HCC T cell chemokines and alleviating tumor T cell rejection. In the preclinical model of HCC, selective inhibition of HDAC-8 increased tumor inhibition of CD8^+^ T cells, and enhanced eradication of HCC by anti-PD-L1 therapy, with good safety (143). Additionally, recent have shown that HDAC-6 inhibits helper T 17 cells (Th17) that produce interleukin-17 (IL-17), thereby inhibiting the antitumor immune response. Adoptive transfer of HDAC-6-deficient Th17 cells can increase IL-17A in the HCC TME, thereby enhancing the anti-tumor response mediated by CD8^+^ T cells. This suggests that HDAC-6 inhibitors can enhance the effect of immunotherapy in an IL-17A-dependent manner. Interestingly, this process also increased the expression of PD-1, making advanced HCC sensitive to ICIs and showing a strong synergistic effect (145). In addition to cytokines, microRNAs (miRNAs) are also important regulatory factors in the HCC microenvironment. It has been mentioned that HDACis may activate NK cells by increasing the expression of NKG2D ligand MICB and exerting antitumor effects. Recent studies have shown that HDACis also facilitates this process in HCC by inhibiting a miRNA called miR-889. miR-889 is considered to be a new MICB-targeting miRNA. Overexpression of miR-889 can significantly inhibit the mRNA and protein expression of MICB in HCC cells, and reduce the cytotoxicity mediated by NK cells. After the use of sodium valproate to inhibit HDACs, HCC cells showed down-regulation of miR-889 and increased sensitivity to NK cells (146).

Most HDACi drugs are approved for the treatment of hematologic tumors, such as PTCL, with a low mutation rate. HCC has been described as having a higher mutation load than most hematological malignancies, suggesting that HDACis have great potential to overcome the immune evasion of HCC. Given the significant obstacles to the development of novel anti-tumor drugs, combining HDACis with immunotherapy is an excellent option to enhance the effectiveness of existing treatment strategies. HDACis can regulate the immunogenicity and TME of HCC tumor cells in a variety of ways, and cope with tumor heterogeneity. Moreover, the effects of HDACis on immunotherapy result in a lower dose, which reduces cytotoxicity and adverse drug reactions (35). Generally, the use of HDACis to synergistically enhance HCC immunotherapy is a multi-mechanism strategy with good application prospects.

## 5 The potential applications of nano-based drug delivery system in HCC

Extensive research has been carried out to find mechanisms involved in the pathogenesis of HCC to develop novel strategies for diagnostic and therapeutic for the past few years. Nanotechnology has significantly affected the medical field by applying nanostructure to achieve specific therapeutic functions and improve medical limitations (165, 166). In this respect, nanotechnology provides huge opportunities in the diagnosis and treatment of HCC, which can target selectivity and specificity, and effectively achieve sufficient dosage in targeted tumor areas without adverse effects or minimal damage to normal cells (167). HCC has the characteristics of hypoxia, vascular leakage, specific receptors and acidic micro-environment, which can be recruited as targeting agents or by designing controlled delivery systems (168). Nanomedicine-based therapeutics have shown the potential to tackle the dilemma of the side effects of conventional chemotherapeutics, and a large number of nanomedicine-based therapeutics are under development for the treatment of HCC (38).

**Figure 3:**
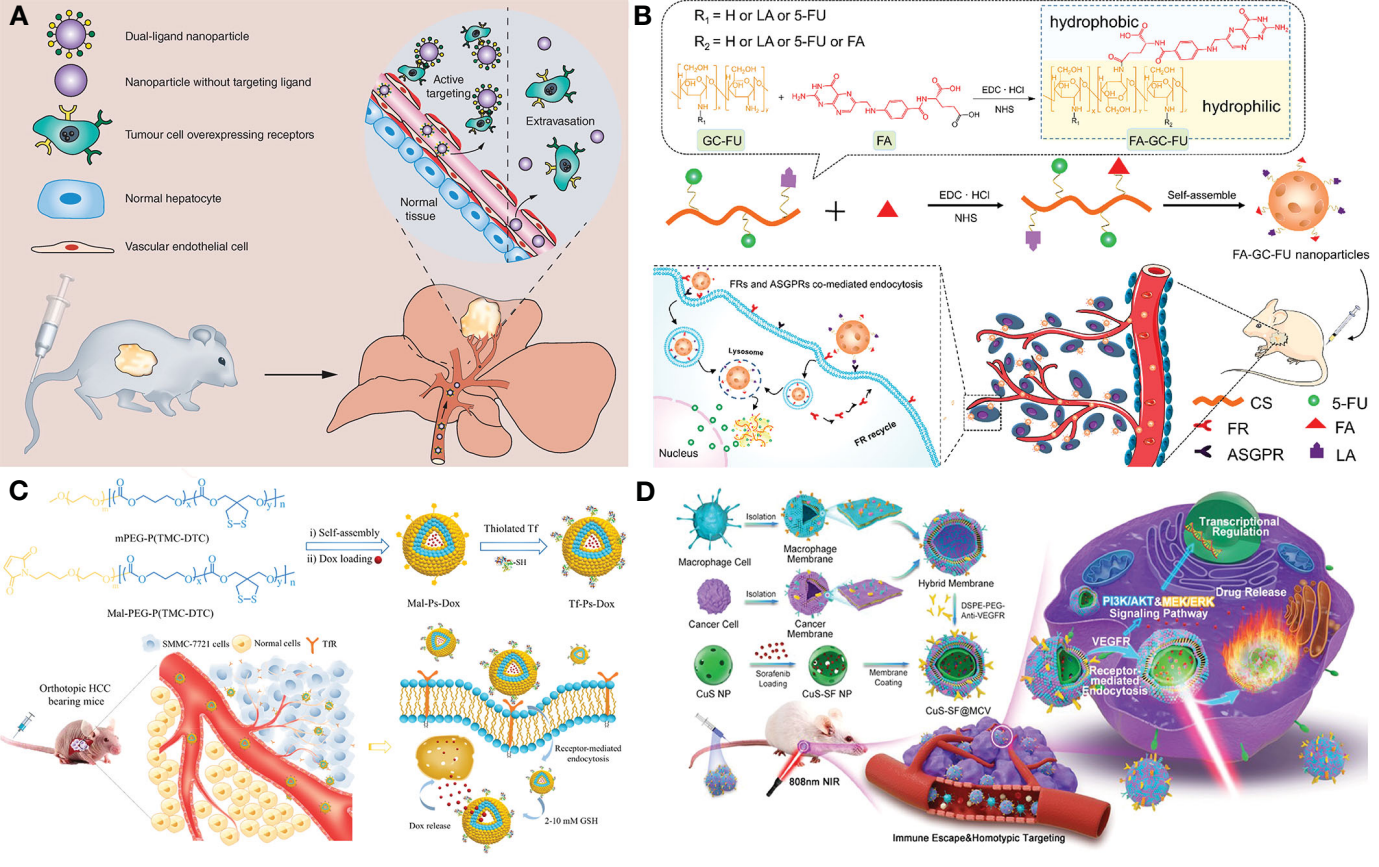
Applications of nano-based drug delivery systems in HCC. **(A)** Schematic representation of the dual-ligand glycyrrhetinic acid and galactose-modified chitosan nanoparticles with dual-ligand targeting hepatoma cells after intravenous administration in tumor-bearing mice model, through enhanced permeability and retention effect and active targeting between lactobionic acid and glycyrrhetinic acid on the nanoparticles and their receptors on hepatoma cells. Adapted with permission from ref (171). Copyright 2020, Future Medicine Ltd. **(B)** Schematic illustration of the facile synthesis of FA-GC-FU and its self-assembled micelle NPs with dual-targeting ligands of FA and LA for hepatoma-targeted delivery of 5-FU. Adapted with permission from ref (173). Copyright 2020, American Chemical Society. **(C)** Schematic illustration of the preparation of transferrin-guided, reduction-responsive and reversibly cross-linked polymersomal doxorubicin (Tf-Ps-Dox), and the targeted therapy of orthotopic hepatocellular carcinoma of Tf-Ps-Dox *in vivo*. Adapted with permission from ref (174). Copyright 2019, Elsevier. **(D)** Schematic illustration of the generation of macrophage−cancer hybrid membrane-coated, sorafenib-loaded and anti-VEGFR-modified CuS NPs for PTT against hepatocellular carcinoma. Adapted with permission from ref (175). Copyright 2020, Elsevier.

Generally, the therapeutic agent and a delivery system containing nano-carriers, targeting moiety, and stimuli-responsive units are the key components of designing a novel therapeutic (169, 170). As nano-carriers, organic nanoparticles (NPs) like dendrimers, polymeric NPs, Lipid-based NPs, Nanogels, and inorganic NPs such as hollow copper sulfide NPs, AgNPs, Bi2S3 NPs, quantum dots (QDs), carbon nanotubes, graphene-based nanomaterials have been proved to be feasible in HCC treatment (168). In the drug delivery system, molecularly targeted strategies for nano-drug mainly comprise passive targeting and active targeting. Passive targeting generally contributes to the EPR effect which allows the NPs to selectively accumulate in the tumor. While, active targeting enables therapeutic agents to be delivered to tumors in a highly specific and efficient manner using different targeting moieties. It mainly works based on recognition between the targeting agent immobilized on the NP surface and the over-expressed targeting agent receptor on the tumor cell’s surface. Large amounts of targeting agents such as small molecule targeting ligands (glycyrrhetinic acid (171), folate, etc.), proteins (transferrin, GPC3 (172), etc.), antibodies (anti-GPC3 antibody, anti-VEGFR antibody, etc.), aptamers (TLS 9a aptamer, EpCAM-specific aptamer, etc.) and peptides (SP94 oligopeptide, etc.) had been reported for HCC therapy. Li et al. designed 5dual-ligand glycyrrhetinic acid and galactose-modified chitosan NPs by using the ionic gelation method as novel hepatoma-targeted drug delivery systems to further improve the targeting capability to HCC (**Figure 3A**). The dual-targeted NPs conquered the unsatisfactory targeting capacity and uptake efficiency of the single-ligand modified drug delivery system and represented an effective and safe drug delivery system for targeted therapy of HCC (171). Further, Xiang et al. developed a facile yet efficient strategy toward dual-targeting ligand-functionalized NPs for precise HCC therapy and potential clinical translation to solve the problems of sophisticated chemical design, multi-step synthesis and purification procedures of most reported NPs with dual-targeted properties (**Figure 3B**). Folate (FA) was introduced as a hydrophobic and targeting component to a hydrophilic macromolecular prodrug (galactosylated chitosan-5-fluorouracil acetic acid (GC-FU)) to afford FA-GC-FU formulation that can self-assemble into NPs without the necessity of physical cross-linking. The FA-GC-FU NPs can target the over-expressed folate receptors (FRs) and asialoglycoprotein receptors (ASGPRs) on the surface of HCC cells, leading to greater targeting efficiency for enhanced therapeutic efficiency of HCC *in vitro* and *in vivo* (173). To provide a potent and low-toxic treatment modality for HCC, transferrin-guided polymersomal doxorubicin (Tf-Ps-Dox) was fabricated with controlled transferrin density, small size, and high drug loading through ligand postmodification strategy by Wei et al. (**Figure 3C**). The Tf-Ps-Dox NPs resulted in up to three-fold more accumulation and longer survival time than non-targeted Ps-Dox and clinically viable liposomal Dox (Lipo-Dox) (174). Biomimetic NPs coated with cell membranes have been widely concerned in targeted anti-tumor therapy due to the enhanced biocompatibility and specificity for homotypic cells. Ji et al. constructed cancer cell-macrophage hybrid membrane-coated hollow CuS NPs encapsulating sorafenib and surface modified with anti-VEGFR antibodies (CuS-SF@CMV) (**Figure 3D**). The CuS-SF@CMV NPs enhanced synergistic photothermal therapy (PTT) and chemotherapy against HCC owing to their immune evasion, tumor cell targeting and drug loading capacities, along with an inherent photo-thermal conversion ability (175). Compared with antibodies, aptamers with the advantages of low molecular weights and lack of immunogenicity show more stability, low cost and equal binding affinities, making them be used as promising targeting moiety candidates. Chakraborty et al. compared the therapeutic potential of phosphorothioate-modified TLS 9a aptamer (L5)-functionalized drug nano-carrier (PTX-NPL5) with other nano-carrier formulas, including previously reported HCC cell-targeting aptamers and non-aptamer ligands functionalized NPs. The results indicated that PTX-NPL5 had the highest potency in inducing selective apoptosis in neoplastic hepatocytes *via* a mitochondrial-dependent apoptotic pathway and did not produce any notable toxic effects in healthy hepatocytes, thus unveiling a new and safer option in targeted therapy for HCC (176).

Although ICIs have shown significant promise for cancer treatment, there are still challenges with efficacy, patient variability and off-target effects when immunomodulators are used (177). Immunomodulators like proteins have limited delivery potential when administered freely. Study has indicated that NPs have the potential to significantly improve delivery by protecting immunomodulators and enhancing their interaction with immune cells (178). Thus, nanomedicines-based immunotherapy has recently received widespread attention as a newly introduced strategy for tumor treatment (179–181). As an anticancer immune-boosting strategy, checkpoint inhibitors are typically monoclonal antibodies that target PD-1, PD-L1 or CTLA4. However, the usage of free antibodies is limited by stability concerns. To improve these therapies by enhancing efficacy and reducing side effects, NPs have been utilized for both monoclonal antibody (anti-PD-1) and small interfering RNAs (siRNAs) delivery, which disrupt immune checkpoints (182, 183). Immunogenic cell death (ICD) is one type of cell death that causes an activation of the immune response (184). Many studies have revealed that drugs that are able to induce ICD are of great significance for cancer therapy (184). Metallic material-derived NPs usually have photothermal therapy (PTT) and photodynamic therapy (PDT) effects, which not only can be used as photosensitizer, but also have great potential for cancer immunotherapy due to ICD. For example, Dong et al. designed a multifunctional FA-CuS/DTX@PEI-CpG NPs (FA-CD@PP-CpG) for synergistic PDT, PTT and docetaxel (DTX)-enhanced immunotherapy (185). FA-CD@PP-CpG can improve immunotherapy effects, such as promoting infiltration of CTLs, suppressing myeloidderived suppressor cells (MDSCs) and enhancing antitumor efficacy on 4T1-tumor-bearing mice. Chemotherapeutic agents such as platinum-based drugs and Dox were identified that not only induce cell apoptosis, but also trigger ICD in tumor cells, leading to activated cytotoxic T cells mediating the anti-cancer immune responses (186). Zhu et al. (180) encapsulated Dox and PD-L1 siRNA (siPD-L1) into block copolymer PEG-PLA (NPDox/siPD L1) to evaluate the effects of Dox on the ICD in the PD-L1 knockdown tumor cells and tumor-bearing animal models. The results demonstrated that the treatment of NPDox/siPD-L1 significantly increased the ICD induction in the HCC cells, supporting the adjunctive role of blocking PD-L1 in the augment of ICD. Additionally, *in vivo* study supported that treatment of NPDox/siPD-L1 significantly inhibited tumor growth.

Epigenetic changes alter the TME by changing gene expressions and silencing tumor-suppressor genes. Hence, DNA methylation and histone modifications are the potential therapeutic targets in cancer therapy (187). However, current epigenetic drugs in cancer therapy are restricted by poor bioavailability, undesired side effects and cytotoxicity to normal tissues (188). Drug delivery system provides the opportunity to overcome the above limitations and improve therapeutic efficacy, owing to delivering high concentration of drugs to the tumor tissue with minimal side effects to healthy tissue. Meanwhile, the integration of two or more anti-tumor therapeutic methods has been proven to improve the therapeutic efficacy compared to the mono-therapy approaches (189, 190). Ruttala et al. developed a transferrin-anchored albumin nanoplatform with PEGylated lipid bilayers (Tf-L-APVN) for the targeted co-delivery of paclitaxel and vorinostat in solid tumors (191). Paclitaxel is an important chemotherapeutic drug with a broad spectrum of activity against multiple solid tumors. However, high toxicity, poor aqueous solubility and poor biodistribution restricted its therapeutic efficacy (192). As an HDACi, vorinostat plays a crucial role in epigenetic transcriptional regulation. The co-loading of paclitaxel and vorinostat could effectively modify the pharmacokinetics and toxicity profiles, control the release of drugs and maintain synergistic drug ratios for maximum therapeutic benefits. The Tf-L-APVN significantly enhanced the synergistic effects of paclitaxel and vorinostat on the proliferation of HepG2 cancer cells and displayed excellent anti-tumor efficacy in HepG2 tumor-bearing mice, making it great potential for HCC therapy.

The above-mentioned researches proved that nanomedicine is one of the novel strategies to help the generation of new therapeutic procedures for HCC. However, challenges and drawbacks in different nanostructures have restricted their applications in the clinic, resulting only a few nano-carriers have successfully entered clinical trials for HCC therapy. Physicochemical characteristics of nanomaterials including size, composition, structure, surface modifications, charge, porosity and aggregation behavior are one of the main challenges, and so, reproducible standards are necessary for improving the quality assessment of nanomaterials. Safety and biocompatibility concerns are also challenges in the translation of nanomedicine products to the clinic due to triggering adverse responses. Although the biomimetic multifunctional nanostructures using different biological compartments such as cell membranes or whole cells have been utilized to overcome the limitation, refining and standardizing requirements for the approval of nanomaterials are also necessary. Additionally, the complexity of the TME brings challenges for drug delivery. To introduce nanomedicine as an extraordinary tool for HCC therapy, more efforts should be made to investigate the easy routes to synthesize therapeutic nanomaterials and ensure their biosafety, cytotoxicity and drug efficiency, besides, multidisciplinary collaboration of different scientific areas is still needed to fully address all challenges.

## 6 Conclusion and perspectives

HCC is a malignant tumor with high morbidity and mortality, which seriously threatens the health of people all over the world. Immunotherapy has opened a new direction for HCC treatment and is gradually transforming the management of HCC patients. However, due to the suppressed tumor immune microenvironment of HCC, some patients are not sensitive to immunotherapy, which hinders the application and development of HCC immunotherapy. Here, we summarize the roles of HDACis, a class of epigenetic regulatory drugs, in enhancing HCC immunotherapy. HDACis have been shown to exhibit superior immunomodulatory capacity in a variety of tumors, including HCC, and have strong tumor suppressor function in conjunction with immunotherapies such as ICIs. The specific mechanisms include enhancing tumor immunogenicity and regulating TME. These results indicate that HDACis are an excellent adjunct drug for immunotherapy, and the two drugs have a stronger synergistic effect while playing their respective anti-tumor functions. However, the choice of drugs for HDACis is a challenge that combination therapy has to face, which is related to the multipotency of HDACi. On the one hand, HDACs can regulate a variety of non-histone targets, and inhibition of one HDAC may lead to multiple outcomes. On the other hand, some HDACis lack selectivity (pan-HDACis) and can inhibit multiple HDACs, which may lead to toxicity to healthy cells. Furthermore, the dosage of HDACis and the interaction between drugs in combination therapy must be carefully considered. Further studies should focus on synthesizing more selective HDACis and trying better combination therapies to reduce possible adverse effects. In addition to drug combination therapy strategies, nanomaterial-based drug delivery systems also open up new directions for improving the therapeutic efficacy of HCC. Based on its advantages of good stability, good targeting, special physicochemical properties and biological effects, NDDS can effectively overcome the drug resistance mechanisms of some tumors, and has achieved impressive results. How to ensure the biosafety of the drug delivery systems, effectively control the cost, and develop uniform nanomedicine application standards will be the main challenges faced by NDDS in HCC treatment.

## Author contributions

YL and YZ designed this study. CS, ML and YD drafted the manuscript. CS performed drawing and organization of figures. YL, YZ, CS, ML, YD, XJ, XH and FX revised the manuscript. All authors read and approved the final manuscript. All authors contributed to the article and approved the submitted version.
